# Werewolf, There Wolf: Variants in *Hairless* Associated with Hypotrichia and Roaning in the Lykoi Cat Breed

**DOI:** 10.3390/genes11060682

**Published:** 2020-06-22

**Authors:** Reuben M. Buckley, Barbara Gandolfi, Erica K. Creighton, Connor A. Pyne, Delia M. Bouhan, Michelle L. LeRoy, David A. Senter, Johnny R. Gobble, Marie Abitbol, Leslie A. Lyons, 99 Lives Consortium

**Affiliations:** 1 Department of Veterinary Medicine and Surgery, College of Veterinary Medicine, University of Missouri, Columbia, MO 65211, USA; buckleyrm@missouri.edu (R.M.B.); Barbara-Gandolfi@idexx.com (B.G.); erica-creighton@idexx.com (E.K.C.); cap998@mail.missouri.edu (C.A.P.); deliabouhan10@gmail.com (D.M.B.); leroymi@missouri.edu (M.L.L.); senterd@missouri.edu (D.A.S.); 2 Veterinary Allergy and Dermatology Clinic, LLC., Overland Park, KS 66210, USA; 3 Tellico Bay Animal Hospital, Vonore, TN 37885, USA; jrgobblevet@gmail.com; 4 NeuroMyoGène Institute, CNRS UMR 5310, INSERM U1217, Faculty of Medicine, Rockefeller, Claude Bernard Lyon I University, 69008 Lyon, France; marie.abitbol@vetagro-sup.fr; 5 VetAgro Sup, University of Lyon, Marcy-l’Etoile, 69280 Lyon, France

**Keywords:** atrichia, domestic cat, *Felis catus*, fur, *HR*, naked

## Abstract

A variety of cat breeds have been developed via novelty selection on aesthetic, dermatological traits, such as coat colors and fur types. A recently developed breed, the lykoi (a.k.a. werewolf cat), was bred from cats with a sparse hair coat with roaning, implying full color and all white hairs. The lykoi phenotype is a form of hypotrichia, presenting as a significant reduction in the average numbers of follicles per hair follicle group as compared to domestic shorthair cats, a mild to severe perifollicular to mural lymphocytic infiltration in 77% of observed hair follicle groups, and the follicles are often miniaturized, dilated, and dysplastic. Whole genome sequencing was conducted on a single lykoi cat that was a cross between two independently ascertained lineages. Comparison to the 99 Lives dataset of 194 non-lykoi cats suggested two variants in the cat homolog for *Hairless* (*HR*) (*HR* *lysine demethylase and nuclear receptor corepressor*) as candidate causal gene variants. The lykoi cat was a compound heterozygote for two loss of function variants in *HR*, an exon 3 c.1255_1256dupGT (chrB1:36040783), which should produce a stop codon at amino acid 420 (p.Gln420Serfs*100) and, an exon 18 c.3389insGACA (chrB1:36051555), which should produce a stop codon at amino acid position 1130 (p.Ser1130Argfs*29). Ascertainment of 14 additional cats from founder lineages from Canada, France and different areas of the USA identified four additional loss of function *HR* variants likely causing the highly similar phenotypic hair coat across the diverse cats. The novel variants in *HR* for cat hypotrichia can now be established between minor differences in the phenotypic presentations.

## 1. Introduction 

Domestic cats have been developed into distinctive breeds during the past approximately 150 years, since the first cat shows were held in the late 1800’s [1,2,3]. Many breeds have proven to be genetically distinct [4,5] but also suffer from inbreeding and founder effects, inadvertently becoming important biomedical models for human diseases. Over 72 diseases/traits caused by at least 115 mutations have been discovered in cat breeds (https://omia.org/) [6,7]. To produce novel breeds, cats have been selected mainly for aesthetic, fur and hair coat traits since the phenotypes can be easily recognized by cat enthusiasts, the unique appearance leading to a new breeding program. A majority of breeds were developed after the World Wars and several are defined by interesting coat DNA variants, such as the Cornish rex [8], Devon rex, sphynx [9], and the Selkirk rex [10,11]. These coat mutations are innocuous in the cat, but the same genes for atrichia and hypotrichia cause ectodermal dysplasias in humans [12,13,14,15] and other species [16,17,18,19,20,21,22]. However, some cat coat and fur types are associated with maladies. The *FOXN1* variant that causes a hypotrichosis in cats is associated with a health condition and shortened life expectancy in the Birman breed [23]. The *White* locus variant in *KIT* has pleiotrophic effects in ocular tissues and is associated with deafness [24]. Albinism and temperature-sensitive variants (*c* (albino), *c^b^* (Burmese), and *c^s^* (Siamese)) in *tyrosinase* (*TYR*) [25,26], the *Color* locus in cats, are associated with disruption of the optical chiasma, leading to strabismus and nystagmus [27]. However, overall, a majority of cat fur types and coat colors have few detrimental health effects.

**Figure 1:**
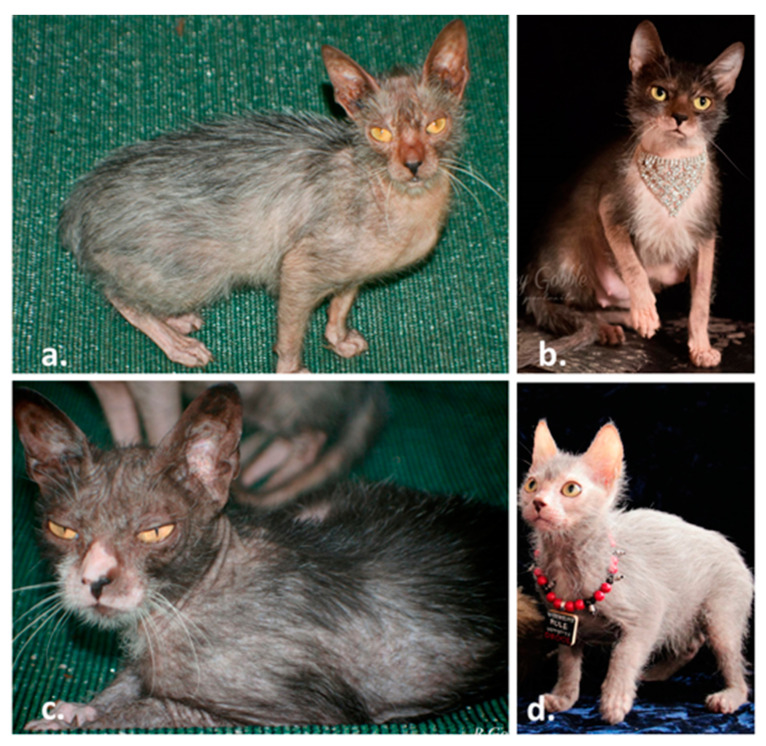
**The lykoi cat breed.** Lykoi breed founders from independent sightings identified in 2010. (**a**) Virginia lineage (c.3389insGACA), (**b**) Missouri lineage (c.1255_1256 dupGT), (**c**) Tennessee lineage (c.1255_1256dupGT), (**d**) Canadian lineage (c.2593C>T). Solid black is the preferred coloration as the roaning of the white hairs is more distinctive. Note sparse hair on the lower limbs. (Images courtesy of Brittney Gobble).

A recently developed breed of cat, termed the lykoi (Figure 1), presents a unique form of hypotrichia [28]. Lykoi have a significant reduction in the average numbers of follicles per hair follicle group as compared to domestic shorthair cats, a mild to severe perifollicular to mural lymphocytic infiltration in 77% of observed hair follicle groups, and the follicles are often miniaturized, dilated, and dysplastic. Individual hairs of the coat are either normal coloration or all white, producing a roaning effect. The undercoats are sparse. Lykoi owners have genotyped their cats for all the known cat fur type mutations, including variants in *KRT71*, which cause the hairless sphynx breed, Devon rex [9] and Selkirk rex [11] curly hair, and none of these variants are present in the lykoi cats. The breeding program was established in 2011 by a veterinarian who has constantly monitored health in the cats [29]. No health concerns have been identified in the lykoi other than the lymphocytic mural folliculitis.

Whole genome sequencing (WGS) has proven a successful genetic approach for the identification of causal gene variants for several phenotypes and diseases in the domestic cat [30,31,32,33]. This study used WGS to identify the causal gene variant(s) for the lykoi presentation in the domestic cats.

## 2. Materials and Methods

### 2.1. Ethics Statement

All procedures performed in studies involving animals were in accordance with the ethical standards of the University of Missouri (MU) institutional animal care and use protocol 8701 and 8313. All samples were collected with informed owner consent.

### 2.2. Lykoi Samples

Samples for DNA isolation from the lykoi cats were provided voluntarily with the permission of the owners as either whole blood EDTA or buccal swabs. Approximately 171 samples were collected between 2013 and 2019 from 11 different owners. These samples included 57 unrelated cats (new potential founders) with similar phenotypes. DNA was isolated by organic methods [34] or using DNAeasy kits (Qiagen, Valencia, CA, USA) according to the manufacturer’s protocol. To develop the French pedigree, from the breeder/owner reported parentage of submitted cats, parentage was verified with a panel of feline-derived short tandem repeats (STRs) as previously described [35]. STR fragment sizes were determined using STR and analysis software [36]. 

### 2.3. Whole Genome and Variant Calling

A single lykoi cat was subjected to whole genome sequencing as previously described [32]. Illumina paired-end sequencing was conducted to produce approximately 30× coverage. The selected cat was an F1 from the mating of two independently discovered foundation lineages from Virginia and Tennessee. The sequence was included in the 195-cat analysis of the 99 Lives cat genome sequencing project and submitted to the NCBI short read archive under BioProject: PRJNA308208, PRJNA288177; BioSample: SAMN05980355. Moreover, all cats for the 195-cat analysis are publicly available through the NCBI short read archive under the accessions shown in Supplementary File 1. For the 195-cat analysis, reads were mapped to Felis_catus_9.0 [37] and assigned to read groups using BWA-MEM from Burrows–Wheeler Aligner version 0.7.17 [38]. Duplicate reads were marked using MarkDuplicates from Picard tools version 2.1.1 (http://broadinstitute.github.io/picard/), with OPTICAL_DUPLICATE_PIXEL_DISTANCE set at 2500. Genome Analysis Toolkit version 3.8 (GATK 3.8) was used to further process the sequence data [39]. Indel realignment was performed with RealignerTargetCreator and IndelRealigner [39] and SNPs, and Indels were called using HaplotypeCaller in gVCF mode (-ERC GVCF) [40]. The gVCFs were combined into groups of ~20 individuals using CombineGVCFs and were genotyped simultaneously using GenotypeGVCFs. Throughout, Samtools version 1.7 sort, index, view, and cat functions were used to process BAM files between individual tasks [41]. Together these processes produced a single VCF comprised of 195-cats for downstream analysis. Code used to process individual genomes is publicly available on github (https://github.com/mu-feline-genome/github-lewis/blob/master/map_libraries.slurm.sh). DNA variants were viewed, filtered and annotated using VarSeq (Golden Helix, Boseman, MT, USA) with the Ensembl release 98 Felis_catus_9.0 genome annotation [42]. VarSeq annotation is similar to Variant Effect Predictor (Ensembl VEP), using the same sequence ontology. Candidate variants were considered to be homozygous or compound heterozygous in the same gene in the lykoi cat and not present in any other cat of the 99 Lives cat database. Only variants that caused high to moderate severity effects (frameshift, nonsense, splice donor–acceptor, splice region, and missense variants) on the protein were considered and variants with high severity and within candidate genes were prioritized. Sequencing primers were developed for candidate variants as previously described [9] for the homolog of *HR* using sequences NCBI Accessions: XM_023252512.1, XM_011281452.3 (Supplementary Table S1).

### 2.4. Hairless (HR) Genotyping and Sequencing

The two *HR* frameshift variants, including an exon 3 c.1255_1256dupGT, and the exon 18 c.3389insGACA, were identified by the WGS analyses. These variants were validated in the WGS cat by Sanger sequencing (Supplementary Table S1) A mass spectroscopy assay was designed as previously described [32] to genotype the identified variants in pedigree A (Supplementary Figure S1) and the additional cats, using the Agena Bioscience iPLEX Gold Genotyping reagent set (Agena Bioscience Inc., San Diego, CA, USA) (Supplementary Table S2). Products were genotyped with the MassARRAY System with Nanodispenser RS1000 (Agena Bioscience Inc., San Diego, CA, USA).

Not all ascertained cats with similar hair coats had the WGS identified variants, therefore the coding regions of *HR* were Sanger sequenced in each additional founder cat (Supplementary Table S1). PCR and thermocycling conditions were conducted as previously described [43]. The variants for the cats in the pedigree B (Supplementary Figure S2) were also genotyped by Sanger sequencing.

## 3. Results

### 3.1. Lykoi Samples

Over 100 cats were ascertained for the lykoi project and were used to develop two pedigrees of the cats segregating for the lykoi phenotype (Supplementary Figures S1 and S2). The relationship of the cats of Pedigree B was confirmed by STRs (data not shown). Sixty-seven cats formed an extended pedigree “A” by crossing three different lineages (Tennessee, Virginia and Texas) (Supplementary Figure S1) and a smaller pedigree “B” was obtained from a French lineage of cats (Supplementary Figure S2). Overall, cats were identified from 16 foundation lines, ascertained from 14 diverse regions in the USA, Canada and France. Two supposed founder lineages were independently ascertained from Florida, California and France, each (Table 1, Figure 1).

### 3.2. Whole Genome Sequencing

**Figure 2:**
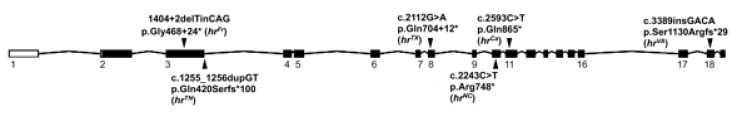
*Hairless (HR)* gene and variants in the lykoi breed. Representation of Ensembl transcript ENSFCAT00000012982 HR—202. Genomic location of the identified variants associated with the Hairless phenotype in the lykoi breed are indicated by triangles. Presented are the coding positions and alteration, the protein alteration and the allele designation. Untranslated regions (UTRs) are presented as open boxes, translated exons are solid. Exon number indicated below boxes. Two of the identified variants disrupt a splicing site (c.1404+2delTinsCAG and c.2112G>A) while all other variants (c.1255_1256dupGT, c.2243C>T, c.2593C>T and c.3389insCAGA) are predicted to produce a truncated protein product.

The selected cat for the WGS represented two founder lineages (Supplementary Figure S1) and a mean of 48.4^×^ genomic sequence coverage was produced for the sequenced cat. Approximately 558 variants within or near coding regions were identified as heterozygous and unique to the lykoi cat. Seventeen were loss of function variants and 154 were missense variants (Table 2, Supplementary File 2). Only one gene was identified with variants that caused highly severe effects on the protein. The two variants in the cat homolog of *Hairless* (*HR*), *HR* *lysine demethylase and nuclear receptor corepressor* (cat chromosome B1:36038754–36052521), were considered the highest priorities as both variants have severe effects and supported the suspected compound heterozygosity in the sequenced lykoi cat. Additionally, *HR* is a known gene causing atrichia in mice [44] and humans [45]. The lykoi cat was a compound heterozygote for two loss of function variants in *HR* transcript (*HR*-202 ENSFCAT00000012982.5); specifically, an exon 3 c.1255_1256dupGT (chrB1:36040784), which should produce a stop codon at amino acid 420 (p.Gln420Serfs*100) in the Tennessee lineage and is designated *hr^TN^* allele and, an exon 18 c.3389insGACA (chrB1:36051556), which should produce a stop codon at amino acid position 1130 (p.Ser1130Argfs*29) in the Virginia lineage and is designated *hr^VA^* allele (Figure 2). These two identified frameshift variants were confirmed by direct Sanger sequencing in the cat submitted for WGS and the presented positions are for the newest cat genome assembly Felis_Catus_9.0 (GCF_000181335.3/). The lykoi phenotype segregated concordantly with each loss of function variant across the pedigree developed from the Virginia and Tennessee lineages (Supplementary Figure S1). Cats with the lykoi hair coat in these lineages were either homozygous for one of the two loss of function variants or compound heterozygous for both loss of function variants.

WGS data also revealed additional *HR* variants. There was one synonymous variant (p.Val1129=), two missense variants (p.Lys433Asn and p.Ser1130Arg), 14 intronic variants, and one 3′ UTR variant. One of the missense variants was an exon 3 c.1299A>C, suggesting a p.Lys433Asn amino acid change of a positively charged lysine to a polar and uncharged asparagine. The p.Lys433Asn was a common variant with an allele frequency of 0.57. Conversely, the p.Ser1130Arg variant was heterozygous in only one other cat in the 99 Lives dataset (Table 2, Supplementary File 3). Heterozygous only splice region variants were identified in the 99 Lives dataset and were unique to Bengal cats, hence perhaps of Asian Leopard cat (*Prionailurus bengalensis*) origin. 

### 3.3. Lykoi Variants in Other Lineages

The *HR* variants discovered using WGS (c.1255_1256dupGT and c.3389insGACA) were absent from other lykoi cats from different lineages, suggesting multiple causative gene variants for the phenotype. To identify additional lykoi variants, direct sequencing of the coding region of *HR* was performed on lineage founders. Four additional variants were identified (Figure 2, Table 1). Firstly, an exon 3 splice variant c.1404+2delTinsGT (chrB1:36040933) was identified in a cat from France Pedigree B and is designated *hr^Fr^* allele (Figure 2, Supplementary Figure S2). This variant should extend and change the reading frame, including an additional 24 amino acids in the aberrant protein before a stop codon is recognized. Alternatively, a cryptic splice site may be used from within intron 4. Secondly, an exon 8 variant at c.2112G>A (chrB1:36045776) was identified in seven different lineages as homozygous, including a cat from France and is heterozygous in a suspected obligate carrier from Florida. This variant likely disrupts the splice donor allowing read-through for an additional 12 amino acids until a stop codon is encountered and is designated *hr^TX^* allele. Alternatively, a cryptic splice site may be used from within intron 8. Finally, two additional stop codon producing variants were also identified including an exon 10 c.2243C>T (p.Arg748X) (chrB1:36047047) in a cat from North Carolina, designated *hr^NC^* allele, and an exon 11 c.2593C>T (p. Gln865X) (chrB1:36047518) identified in two cats from Tennessee and Canada and is designated *hr^Ca^* allele. Five submitted founder cats had unique variants. The founder cat from Canada had the same variant as one of the submitted founders from Florida. The other cat from Florida had a normal coat but was the offspring of a suspected new lineage. This cat was heterozygous for the exon 8 splice site variant thus, the queen did not have a novel variant. Overall, six likely causal gene variants were identified (Figure 2) in 16 lineages, including seven lineages from the USA covering 11 states and one cat from France sharing the same exon 8 splice site variant. The variants, positions and flanking sequences are presented in Supplementary File 4.

Known crosses of the different foundation lineages supported the causal function of the identified variants. Sixteen cross lineage cats that had the lykoi hair coat were compound heterozygotes, including 16 for the *hr^TN^/hr^VA^ alleles* (Supplementary Figure S1) and compound heterozygous lykoi cats with the *hr^TX^/hr^VA^* alleles in both pedigrees (Supplementary Figures S1 and S2). One of the seven cats with the exon 3 c.1299A>C non-synonymous variant was also homozygous for the exon 10 c.2243C>T (p.Arg748X) stop codon variant and one other cat was homozygous for the exon 8 splice variant, further suggesting this missense variant as non-causal. The cats from France had been cross bred with cats from Italy and the USA, demonstrating the presence of the exon 8 and exon 18 variants. The French pedigree (Pedigree B—Supplementary Figure S2) also segregated for a novel exon 3 splice variant, indicating a novel de novo variant from Europe. 

## 4. Discussion

Although over 50 cat breeds are identified by different cat associations and registries worldwide, fewer than 30 are demonstrated to be genetically distinct [4,5,46]. Novel cat breeds are continually being developed by producing a new breed from crosses with existing breeds, such as the Ocicat and Burmilla, by interbreeding domestic cats with small wild felids, such as Bengals and Savannahs, and by identifying new phenotypic variants in feral populations i.e., novelty selection, such as Devon [47], Cornish [48] and Selkirk rex [10]. Novelty breeds, such as Selkirk rex and Scottish folds, are characterized by novel “breed-defining” variants, retain high genetic variation [4,5,10], but often modify their type but by cross-breeding with established breeds that have the desired structural “look”. For example, the Selkirk rex has strong genetic influences from Persians and British shorthair [10], although the curly coat is a novelty phenotype identified in the past few decades in Northwestern USA [10].

The lykoi is a very recently developed novelty breed with a sparse hair coat and black and white hair roaning, hence named from the Greek term *lycos* for wolf. To maintain diversity in the founding population, the breeders have actively recruited cats with similar phenotypes for the breeding program, resulting in six different “foundation” lineages identified in this study from 16 potential founders. The breed is growing in popularity due to the novelty of the appearance, the lack of concern for health problems and the charismatic name and nature. The breed was accepted for full championship showing by TICA in May 2017 [49].

*Hairless* (*HR*) (a.k.a. *HR* *lysine demethylase and nuclear receptor corepressor*) is one of the earliest mutations identified in mice (MMu Chr14:70554056-70573548) and over 30 phenotypic mutations have been identified, including ~17 that are spontaneous and naturally occurring (MGD) [50]. The hairless mouse [51,52] is an insertion of murine leukemia proviral sequences into intron 6 resulting in aberrant splicing [44,53,54]. The *HR* gene encodes a protein that is involved in hair growth. This protein functions as a transcriptional corepressor of multiple nuclear receptors, including thyroid hormone receptor [55], the retinoic acid receptor-related orphan receptors [56] and the vitamin D receptors [57], and also interacts with histone deacetylases [58]. By modulating the activity of receptors, *HR* plays a critical role in skin function and hair maintenance by regulating both gene expression as well as epithelial stem cells differentiation. The translation of this protein is modulated by a regulatory ORF that exists upstream of the primary ORF, hence, the protein expression regulation is an overall critical element in directing hair growth [59]. The human homolog, *HR*, is on human chromosome 8p21.3; chr8:22114419-22131053. ClinVar lists 187 variants involving *HR*, 117 are limited to the gene and 17 are pathogenic or likely pathogenic mutations in humans [60]. Several *HR* variants are known to cause abnormalities in humans, such as alopecia universalis congenita (OMIM:203655) [55], atrichia with papular lesions (OMIM:209500) [61], which is an alopecia characterized by irreversible hair loss during the neonatal period on all hair-bearing areas of the body followed by the development of papular lesions, and Hypotrichosis 4, (a.k.a.) Marie Unna Type, 1; (OMIM:146550), which is caused by autosomal dominant mutations in the upstream ORF–U2RH [59]. Variants in *HR* in other species are relatively rare, but causal gene variants of hairless are known in sheep [17], atrichia with papular lesions is also identified in macaques [16], and, in dolphins, evolutionary loss has led to *HR* as a pseudogene, leading to hypotrichosis in this mammal [20]. 

Various other genes cause hairless phenotypes, such as, *Keratin 71 (KRT71*) in the sphynx cat breed [9], and *Forkhead Box I3* (*Foxi3*) in Mexican and Peruvian hairless dogs and Chinese crested dogs [19] and Serum/Glucocorticoid Regulated Kinase Family Member 3 (*SGK*) [62,63] in Scottish deerhounds. Hair follicle (HF) morphogenesis during embryogenesis is induced by Wnt/β-catenin signaling that leads to the development of the HF placode [64]. Postnatal HF morphogenesis is regulated by *SGK3* through modulation of β-catenin dependent transcription processes [65]. *Foxi3* has been shown to regulate several aspects of HF development and homeostasis, including stem cell specification during induction [66,67]. Wnt/β-catenin activation is correlated with the presence of nuclear *HR* protein at anagen initiation. Although HR function is not recognized during initial hair morphogenesis, *HR* controls the timing and location of *HF* regeneration via Wnt-signaling [66]. Keratins are highly expressed in the terminally differentiated cells in the growing hair follicle, specifically the inner root sheath (IRS). Thus, the genes demonstrated to cause atrichia in various species are due to disruptions of the interwoven pathways and cascades leading to mammalian hair development and expression.

*HR* in the cat is annotated in Ensembl 98 [42] as ENSFCAG00000012978 B1:36034352-36051895:1. Three transcripts are described containing 17–19 exons, in which exons 17–19 are the variable exons. Three 5′ UTRs are recognized, one as part of the 5′ portion of exon 1. Two transcripts have short 3′ UTRs at the end of exon 18. The variants in this study were annotated with Ensembl 98 transcript *HR*-202 containing 4227 bp that translate to 1184 amino acids from 18 translated exons. Each of the six variants identified in the lykoi cats either cause termination codons at the variant site or cause downstream terminations after an additional 12 amino acids after the exon 8 c.2112G>A variant or an additional 100 amino acids after the exon 3 c.1255_1256dupGT variant, leading to proteins with ~528 − 704 + 12 amino acids. Interestingly, one variant, exon 18 c.3389insGACA (p.Ser1130Argfs*29), while associated with the phenotype, produces an almost full length protein (95%), suggesting the terminal end of the protein is required for normal function.

Several phenotypic traits in cats are heterogeneous, including the variants for the loci *Long*, *Tailless*, and the classic (blotched) pattern of *Tabby*, which are each caused by four different mutations in the genes *FGF5* [68,69], *TBX1* [70], and *LVRN* [71], respectively. Variation in the phenotypic presentations caused by these different variants is undocumented. Only the *TBX1* variants define breeds, the Manx and Cymric, which is a longhaired Manx, the *Long* and *Tabby* variants segregate within and amongst breeds. A few breeds have unique and breed defining variants, such as Scottish folds [72], Selkirk rex [11], Devon rex, and Sphynx [9]. Like the Manx, the lykoi will be a unique breed that segregates for several variants within the same gene, *HR*, that present a similar phenotype (Figure 1). Unlike the Manx variants [70], the variants that cause the hypotrichosis are recessive and do not cause additional health concerns known to date. The only documented abnormality is the sparse haircoat resulting from abnormal follicular development and lymphocytic mural folliculitis. Some variants are not perpetuated as they tend to cause more periodic hair loss, suspected to be associated with sex hormone levels (JRG, personal communication). The lykoi breeders can now use genetic testing to monitor the variants in the population and to realize possible associations with phenotypic differences in compound heterozygotes. Additional haplotype analyses of flanking variants could determine if the eight reported founder lineages with the exon 8 variant are identical by descent or identical by state and represent multiple de novo mutation events at the same site.

## Figures and Tables

**Table 1:** Lykoi founder lineage variants in Hairless (HR) for cats with the hypotrichia presentation.

Founder Lineage	*hr^TN^*	*hr^Fr^*	*hr^TX^*	*hr^NC^*	*hr^Ca^*	*hr^VA^*	*HR* Alleles ^‡^
	Exon 3	Exon 3	Exon 8	Exon 10	Exon 11	Exon 18	
	c.1255_1256 dupGT	c.1404+2delTinsCAG	c.2112G>A	c.2243 C>T	c.2593 C>T	c.3389ins GACA	
WGS F1 TN/VA *	Wt/**dup**		G/G	C/C	C/C	Wt/**ins**	*hr^TN^/hr^VA^*
**Tennessee**	**dup/dup**		G/G	C/C	C/C	Wt/Wt	*hr^TN^/hr^TN^*
**Virginia**	Wt/Wt		G/G	C/C	C/C	**ins/ins**	*hr^VA^/hr^VA^*
**Missouri**	**dup/dup**		G/G		C/C	Wt/Wt	*hr^TN^/hr^TN^*
**France L10, L4**	Wt/Wt	**CAG/CAG**	G/G	C/C	C/C	Wt/Wt	*hr^Fr^/hr^Fr^*
**Texas**	Wt/Wt		**A/A**		C/C	Wt/Wt	*hr^TX^/hr^TX^*
**California 1**	Wt/Wt		**A/A**		C/C	Wt/Wt	*hr^TX^/hr^TX^*
**California 2**	Wt/Wt		**A/A**	C/C	C/C	Wt/Wt	*hr^TX^/hr^TX^*
**France L12**	Wt/Wt		**A/A**	C/C	C/C	Wt/Wt	*hr^TX^/hr^TX^*
**Georgia**			**A/A**				*hr^TX^/hr^TX^*
**South Carolina**	Wt/Wt		**A/A**	C/C	C/C	Wt/Wt	*hr^TX^/hr^TX^*
**Utah**	Wt/Wt		**A/A**		C/C	Wt/Wt	*hr^TX^/hr^TX^*
**Vermont Gonzalo**	Wt/Wt	Wt/Wt	**A/A**	C/C	C/C	Wt/Wt	*hr^TX^/hr^TX^*
**North Carolina**	Wt/Wt		G/G	**T/T**	C/C	Wt/Wt	*hr^NC^/hr^NC^*
**Canada**	Wt/Wt		G/G	C/C	**T/T**	Wt/Wt	*hr^Ca^/hr^Ca^*
**Florida 1**	Wt/Wt		G/G	C/C	**T/T**	Wt/Wt	*hr^Ca^/hr^Ca^*
**Florida 2 ^†^**	Wt/Wt		G/**A**	C/C	C/C	Wt/Wt	*Wt/hr^TX^*
**Protein Change**	**Q420S**	**Splice**	**Splice**	**R748X**	**Q865X**	**S1130R**	

Bolded are the causal variant for each cat. * Cat used for WGS was a cross of two lineages. ^†^ One cat had an unknown phenotype but reported as an offspring from lykoi breedings for a new lineage. ^‡^ Alleles named after the state or country of the cat’s origin in which they were first identified. NC is North Carolina USA, TN is Tennessee USA, TX is Texas USA, VA is Virginia USA, Ca is Canada, Fr is France.

**Table 2:** Unique 99 Lives heterozygous whole genome sequencing variants in a lykoi cat.

Severity	Effect	No.	Genes	Hairless (HR)
High	Frame Shift (LOF) ^†^	13	12 *	2
	Splice donor - acceptor	0	0	0
	Stop gained (LOF)	4	5	0
Moderate	Missense	154	146	62
Low	Splice region	31	30	5
	Synonymous	127	120 ^†^	67
Other	Intronic	118	103	9
	Intergenic & UTR	17	--	7
	Non-coding exon	94	68	0
	Total variants	558	~426 **	152

* Two variants in *HR*, ^†^ including p.Ser1130Arg in *HR*. Three variants in *HR* were unique to the lykoi cat in the WGS comparison to 193 additional cats. ** Total includes undefined transcripts and suspected coding sequences. ^†^ LOF implies loss of function, UTR implies untranslated region (5’ or 3’).

## Supplementary Materials

The following are available online at https://www.mdpi.com/2073-4425/11/6/682/s1, Supplementary Table S1: PCR primers for the analysis of *HR* in cats, Supplementary Table S2: MassAarray primers for the analysis of *HR* in cats, Supplementary Figure S1: USA lineages of lykoi cats–Pedigree A, Supplementary Figure S2: French lineages of lykoi cats—Pedigree B, Supplementary File S1: NCBI accessions for samples used in the 99 Lives 195-cat analysis, Supplementary File S2: Heterozygous variants within or near coding regions unique to the whole genome sequenced lykoi cat, Supplementary File S3: All variants found within *HR* in 99 Lives 195-cat analysis, Supplementary File S4: Genomic positions of all lykoi variants identified along with nearby flanking sequence.
